# A genetics-first approach to understanding autism and schizophrenia spectrum disorders: the 22q11.2 deletion syndrome

**DOI:** 10.1038/s41380-022-01783-5

**Published:** 2022-10-03

**Authors:** Ania M. Fiksinski, Gil D. Hoftman, Jacob A. S. Vorstman, Carrie E. Bearden

**Affiliations:** 1 Department of Psychology and Department of Pediatrics, Wilhelmina Children’s Hospital, University Medical Center Utrecht, Utrecht, The Netherlands; 2 Department of Psychiatry and Neuropsychology, Division of Mental Health, MHeNS, Maastricht University, Maastricht, The Netherlands; 3 Department of Psychiatry and Biobehavioral Sciences, Semel Institute for Neuroscience and Human Behavior, University of California, Los Angeles, CA USA; 4 Program in Genetics and Genome Biology, Research Institute, and Department of Psychiatry, The Hospital for Sick Children, Toronto, ON Canada; 5 Department of Psychiatry, University of Toronto, Toronto, ON Canada; 6 Department of Psychology, University of California, Los Angeles, CA USA

**Keywords:** Psychiatric disorders, Schizophrenia

## Abstract

Recently, increasing numbers of rare pathogenic genetic variants have been identified that are associated with variably elevated risks of a range of neurodevelopmental outcomes, notably including Autism Spectrum Disorders (ASD), Schizophrenia Spectrum Disorders (SSD), and Intellectual Disability (ID). This review is organized along three main questions: First, how can we unify the exclusively descriptive basis of our current psychiatric diagnostic classification system with the recognition of an identifiable, highly penetrant genetic risk factor in an increasing proportion of patients with ASD or SSD? Second, what can be learned from studies of individuals with ASD or SSD who share a common genetic basis? And third, what accounts for the observed variable penetrance and pleiotropy of neuropsychiatric phenotypes in individuals with the same pathogenic variant? In this review, we focus on findings of clinical and preclinical studies of the 22q11.2 deletion syndrome (22q11DS). This particular variant is not only one of the most common among the increasing list of known rare pathogenic variants, but also one that benefits from a relatively long research history. Consequently, 22q11DS is an appealing model as it allows us to: (1) elucidate specific genotype–phenotype associations, (2) prospectively study behaviorally defined classifications, such as ASD or SSD, in the context of a known, well-characterized genetic basis, and (3) elucidate mechanisms underpinning variable penetrance and pleiotropy, phenomena with far-reaching ramifications for research and clinical practice. We discuss how findings from animal and in vitro *s*tudies relate to observations in human studies and can help elucidate factors, including genetic, environmental, and stochastic, that impact the expression of neuropsychiatric phenotypes in 22q11DS, and how this may inform mechanisms underlying neurodevelopmental expression in the general population. We conclude with research priorities for the field, which may pave the way for novel therapeutics.

## Introduction

The 22q11.2 deletion syndrome (22q11DS; OMIM #188400, #192430) is a multi-system disorder caused by a microdeletion of up to 3 Megabases of genomic sequence on the long arm of chromosome 22. Phenotypic manifestations vary between individuals, but most frequently include congenital malformations of the heart and palate, immune deficiencies, and endocrine abnormalities [1]. Of particular relevance to this review, 22q11DS is also associated with multiple brain-related phenotypes that may occur independently, in concert, and in varying degrees of severity [2, 3]. Of these, the elevated risk of schizophrenia spectrum disorders (SSD; ~20–25%) in individuals with 22q11DS is the best known and most widely studied to date. However, the risk of other developmental disorders which manifest earlier in life is also increased in 22q11DS, including intellectual disability (ID; ~45%), attention deficit- hyperactivity disorder (ADHD; ~35%), anxiety disorder (~35%) and autism spectrum disorders (ASD; 10–40%) [3–8].

This narrative review focuses particularly on ASD and SSD in 22q11DS, highlighting both clinical and research perspectives. The focus is chosen not only because of the plethora of research on these conditions, both of which are highly prevalent in 22q11DS, but also because of the increasing number of other rare pathogenic genetic variants identified, which appear to have similarly increased rates of ASD, SSD, or both (e.g., 16p11.2 deletion, 3q29 deletion, 1q21.1 deletion). Compared to these, many of which were identified relatively recently, a focus on 22q11DS has two advantages: first, a longer research history given the recognition of its genetic etiology in the early 1990s [9, 10], the identification of its association with SSD over two decades ago, and with ASD at the turn of the century [11–14], and second, its position as “the most frequent among the rare” recurrent disorders. Indeed, with an estimated prevalence of 1 in 2000–6000 live births, 22q11DS is the most common recurrent pathogenic deletion [1, 2]. Given this, several observations regarding both clinical and preclinical aspects of the neuropsychiatric phenotype of 22q11DS provide valuable insights which may not be easily acquired otherwise, and which likely have relevance beyond 22q11DS.

First, while diagnoses of ASD or SSD remain based on behavioral observations, what distinguishes these diagnoses in 22q11DS from most other patients with these conditions is that in the former, ASD or SSD can be associated with a known genetic basis. Therefore, clinical experience with this population may shed light on the co-occurrence of psychiatric and genetic diagnoses, and possibly, how they can be better integrated.

**Table 1:** Glossary.

Coisogenic mouse—developed in embryonic stem cells of a mouse strain and bred with the same mouse line to maintain the genetic background of wild-type and mutant mice as identical.
Congenic mouse—backcrossing an F2 generation mouse to the breeder for more than 10 generations to saturate the genetic background with alleles of the breeder, thereby minimizing the systematic difference in the flanking regions between wild-type and mutant mice.
Copy number variant (CNV)—a deviation of the number of alleles (typically 2n for autosomes) of genomic sequence (ranging from a few to several millions of basepairs in length). A deletion refers to the loss of one allele (1n), a duplication refers to an increase in copies (3n).
Df1/+ mouse—a common 22q11.2 mouse model with a hemizygous deletion of the Dgs-i-Ufd1l (Df1) region. This is the second largest deletion model in the homologous murine chromosome 16; Df1/+ mice show cardiovascular anomalies like those seen in humans with 22q11.2 deletions, as well as hyperactivity and deficits in prepulse inhibition.
Df(h22q11)/+ mouse—another common 22q11.2 mouse model with a hemizygous deletion of the region containing Dgcr2-Hira genes. Similar phenotype to the Df1/+ mouse above.
DGCR8—DiGeorge syndrome critical region gene 8 is a microprocessor complex subunit in the 22q11.2 locus that is involved in microRNA processing, which regulates gene expression by binding messenger RNAs to silence their translation. DGCR8 mutations in mice disrupt neuronal morphogenesis, synaptic plasticity, and cognitive performance.
LgDel/+ mouse—well-established 22q11.2 mouse model with the largest deletion in the homologous murine chromosome 16. LgDel mice have a hemizygous deletion from Idd to Hira, a region containing 24 genes (5 more genes than the Df1/+ mice). They show similar phenotypes to 22q11DS in humans, including cardiac and parathyroid defects, and deficits in prepulse inhibition and in a visual reversal-learning task.
Penetrance—categorically, penetrance can be defined as the prevalence of a given phenotype among individuals with the same pathogenic genetic variant. For example, the penetrance of schizophrenia for 22q11DS is estimated to be 25%. Alternatively, the dimensional assessment of penetrance is a quantitative measure of phenotypic deviation from the population mean. For example, the effect on intellectual ability of 22q11DS can be expressed as an average left shift of ~30 IQ points, or ~2 standard deviations (SD) compared to the IQ distribution in the general population.
Pleiotropy—phenomenon in which a single genetic locus (or mutation) is associated with more than one phenotypic trait/disorder. For example, certain genes increase risk for both ASD and congenital cardiac malformations. Here, we use the term pleiotropy broadly to refer to the range of phenotypic manifestations of the 22q11.2 deletion.
PRODH—proline dehydrogenase (or proline oxidase) is a mitochondrial enzyme in the 22q11.2 locus that catalyzes proline degradation, which is converted to glutamate. PRODH mutations in humans result in hyperprolinemia and are associated with seizures, motor and cognitive delay, aggression, hyperactivity, stereotypic behaviors, and sleep disturbances.
Research Domain Criteria (RDoC)—this alternative approach to psychiatric nosology posits that measures based on dimensions and observable behaviors (both within and across disease diagnoses) may be more informative than our current diagnostic system about mechanisms underlying neuropsychiatric disorders.
SEPT5—Septin 5 is a gene located in the 22q11.2 locus; it is a member of the septin gene family of nucleotide-binding proteins that is implicated in cytoskeletal organization.
Stochastic influences- variation as an intrinsic feature of biological developmental processes. As a result of stochastic variation, the impact of a genetic variant on a brain developmental program may lead to more than one outcome if given a chance to run more than once.
TBX1—T-Box transcription factor 1 is in the 22q11.2 locus and encodes transcription factors involved in regulating developmental processes. Mutation is believed sufficient to cause most of the physical features of 22q11DS.
Variable expressivity—refers to the variability in the severity of a phenotype when present as a result of a pathogenic genetic variant.
ZDHHC8—zinc finger DHHC-type palmitoyltransferase 8 is a gene in the 22q11.2 locus thought to modulate neurotransmitter systems, including activity-dependent plasticity at glutamate synapses, via palmitoylation.

**Fig. 1:**
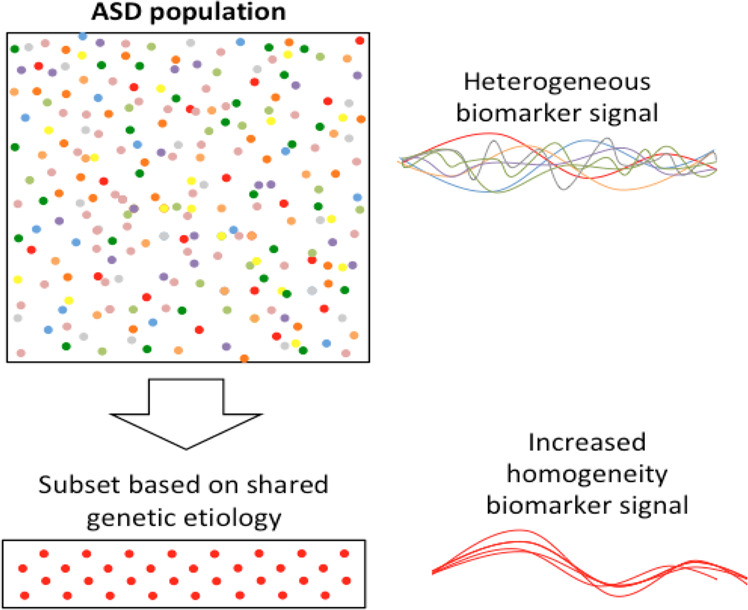
One challenge of biomarker research in a clinical population, such as patients with a specific neurodevelopmental diagnosis (e.g., ASD or schizophrenia), is the underlying etiological heterogeneity (different colored circles). Theoretically, extracting a subset of patients based on a shared genetic origin (red circles only) such as 22q11DS, could reduce heterogeneity, thereby improving the ability to detect meaningful biomarker signals. Notable examples include the highly reproducible neuroanatomic signature of 22q11DS-associated psychosis [169, 156, 159], and emerging evidence for distinguishable ASD profiles in subgroups of individuals with ASD according to genetic etiology [170–173]. Examples of reduced heterogeneity in genetically selected subsets of patients are also observed in other genetic conditions; for example, distinctive electrophysiological brain wave patterns observed in children with ASD related to the 15q11.2-q13.2 duplication compared to children with idiopathic ASD [174], and macrocephaly and gastrointestinal problems in children with ASD related to mutations in *CHD8* [175].

Second, from a research perspective, studying ASD and SSD in the context of 22q11DS offers the advantage of a relative reduction of biological heterogeneity underlying these phenotypes. Consequently, at least theoretically, one may expect to see less signal dilution when studying putative biomarkers (Fig. 1). This also facilitates translational studies, in which experimental manipulations not possible in humans can provide greater insights into underlying biology.

Third, the phenotypic heterogeneity in 22q11DS, observed despite the shared 22q11.2 deletion, is consistent with observations in other populations carrying pathogenic variants [15]. While this heterogeneity may appear incongruous with the previous statement, it is important to note that a phenotypic signal may be increased in a genetically homogeneous subgroup, even though not every individual with the genetic risk factor expresses the phenotype.

As such, the study of neuropsychiatric disorders in the context of 22q11DS allows elucidation of the possible modifying influence of additional factors on phenotypic expression [16, 17]. In addition, given that the genetic diagnosis is increasingly made around birth (or even in utero), there is a valuable opportunity for prospective longitudinal studies to elucidate developmental trajectories of phenotypes such as ASD and SSD, starting early in life [18]. Early identification, prior to the emergence of the full phenotypic manifestations of neuropsychiatric disorders, may also reduce the impact of stochastic influences, thus increasing the signal of the initial effect of the genetic variant on underlying biological mechanisms [19]. This review will discuss the literature from these three perspectives, highlighting the study of 22q11DS as a model for our understanding of the neuropsychiatric phenotypic manifestations observed in a growing list of identified rare pathogenic variants.

## A phenomenological classification in the context of a genetic diagnosis

The exclusive reliance on behavioral characteristics distinguishes psychiatric disorders from most other diagnoses in the medical field, which typically include objectively measurable biomarkers that can be detected by diagnostic examinations and/or laboratory tests. In addition, a psychiatric diagnosis is typically agnostic with regard to etiology.

However, in particular for ASD, in an increasing proportion of cases a genetic basis can be identified [20–23]. This is a new development in the field of psychiatry, with the highest rates observed for ASD (20–30%; [24]), and lower, but slowly increasing, estimates for SSD (5–10%; [25]). The contrast with 20 years ago is stark, when a genetic contribution was identifiable in only about 3% of patients with ASD [24]. These advances in our understanding of the genetic underpinnings of developmental and psychiatric disorders raise a novel question: *How to reconcile the descriptive nosology of psychiatric diagnoses with the identification of specific genetic contributions in growing subsets of patients?*

The use of standard psychiatric diagnostic classification is consistent with the nature and course of the core symptoms of ASD and SSD in 22q11DS, which are similar to those observed in patients with these conditions in the general population [26–28]. However, the connection between 22q11DS and neurodevelopmental and psychiatric disorders is often not made explicit [29–31], which can lead parents and clinicians to assume the child has two unrelated diagnoses. Hence, a logical solution for this would be to integrate psychiatric diagnosis and identified genetic basis into a single diagnostic formulation, when applicable. This would be in line with a recently proposed ‘dyadic approach’ to encompass both phenotypic descriptor and molecular etiology [32]. Applied to psychiatry, this can be achieved by explicitly mentioning that the observed psychiatric phenotype occurs in the context of an identified genetic condition, thereby avoiding the misconception of two independent diagnoses (one psychiatric, one genetic; [32]). The current version of the DSM [33] is not consistent with regard to the use of such specification, proposing it for some, but not all, relevant psychiatric disorders [33]. The objective is not to divide all psychiatric diagnoses by genetic classifications, but rather to provide information on genetic contribution to a disease diagnosis for a subset of patients for whom this is applicable. This has clinical relevance for understanding other related symptoms the patient may experience, as well as course and prognosis of the psychiatric condition. Importantly, the addition of such a specifier does not rule out additional contributing factors, nor does it imply that all individuals with the genetic condition manifest the psychiatric phenotype.

## Genetic heterogeneity

### The value of focusing on a genetically defined subset

The rapid shifts in our understanding of the genetic architecture of developmental neuropsychiatric disorders have led to an emerging picture of substantial etiological heterogeneity. This may partially explain the difficulty identifying biomarkers that can reliably distinguish an entire group of patients with a particular psychiatric condition from controls [34]. The biological heterogeneity of both ASD and SSD, echoing similar findings for other neurodevelopmental disorders such as ID and ADHD, therefore raises the question *whether this heterogeneity is reduced in subsets of individuals with a specific neuropsychiatric diagnosis who share a common genetic basis?* This question is not merely academic, as it may have important consequences for treatment strategies [25], including the potential for precision medicine approaches [35, 36]. Current observations indicate that across a plethora of distinct genetic etiologies, common expressions of behavioral phenotypes can be observed, e.g. cognitive impairment, social-communicative deficits and repetitive behaviors, and/ or positive psychotic symptoms; clusters of these traits suggest categorical diagnoses of ID, ASD or SSD, respectively.

Another largely unanswered question in this regard is: *Where and how in the trajectory from genetic variation to common psychiatric manifestations does this convergence arise* [37]? The elucidation of this issue is complicated precisely by the etiological heterogeneity of patient cohorts in the general population, and the relative rarity of each individual genetic contribution. In this context, the study of individuals with 22q11DS, a high-impact variant associated with several brain-related conditions, may provide valuable insights, because of the increased etiological homogeneity inherent to the selection based on a shared genetic origin (Fig. 1).

The strongly elevated baseline prevalence rates of brain-related disorders including ASD and SSD in the context of 22q11DS—compared to the general population—illustrates how the study of individuals with 22q11DS may serve as a magnifying lens for studies of ASD and SSD, and other brain-related expressions, and their underlying mechanisms in the broadest sense.

### Molecular insights from experimental model systems

#### Animal models

Over the past ~20 years, animal models have begun to show how different mutations within the 22q11.2 locus, or the homologous region in the murine chromosome 16 locus, can result in both distinct and overlapping developmental, cellular, molecular, and behavioral phenotypes. These animal models that seek to link genetic mutations with neurobiological phenotypes and behavioral constructs in a controlled experimental fashion are critical for understanding mechanisms of neuropsychiatric disorder dimensions. Model systems include mouse [38–43]; zebrafish [44, 45], fly (*Drosophila*) [45–47], and worm (*Caenorhabditis elegans*) [45]. This approach of modeling the same genetic defect seen in humans has strong construct validity [48] and avoids the pitfalls of inferring that animal behavior in particular contexts (e.g., immobility during tail suspension) are analogous to human psychiatric disorders [20, 49, 50]. However, given incomplete penetrance and variable expressivity in 22q11DS, these model systems may be more appropriately conceptualized as liability rather than disease models.

The typical ~3 Megabase deletion in the 22q11.2 locus that occurs in roughly 85% of patients with 22q11DS includes more than 40 known protein-coding genes, seven microRNA genes, and 10 non-coding genes, as well as additional predicted coding and non-coding genes [1, 51]. Tbx1 and Dgcr8 are two individual genes within the locus that have garnered attention since they are intolerant to loss of function and have widespread effects on gene regulation; Tbx1 is a transcription factor and Dgcr8 is a protein involved in a microRNA processing complex [52, 53]. Numerous murine models targeting genes in the human 22q11.2 locus have been developed to better understand the contribution of single and multiple 22q11.2 genes to developmental, cell-specific, and behavioral deviations relative to wild-type littermates [54–59]. For example, *Tbx1* heterozygous mice showed significant alterations of myelinated axons in the fimbria, lower mRNA levels of oligodendrocyte-related genes, and postnatal progenitor cells from the subventricular zone produced fewer oligodendrocytes in vitro [60]. These mice also showed selectively slower acquisition of spatial memory and cognitive flexibility. A *Dgcr8* hemizygous deletion model showed deficits in prefrontal cortex projection neuron dendritic spines, as well as reduced progenitor neuron proliferation and neurogenesis in the adult hippocampus [43, 55]. Together, these findings show that haploinsufficiency of 22q11 genes alters specific subsets of neurons and glia during development, and across cortical layers and regions.

The cellular specificity of particular 22q11.2 genes may provide insights into the nature and timing of alterations that underlie specific behavioral phenotypes found in 22q11DS, idiopathic ASD, and SSD. For example, LgDel/+ mice show selective alterations in superficial layer 2/3 pyramidal neuron morphology, similar to the specificity seen in postmortem tissue from SSD subjects [61–66]. In addition, alterations in superficial pyramidal and PV neurons in 22q11DS liability models parallel findings in postmortem studies of idiopathic SSD and ASD [67–69]. At a circuit level, alterations in excitatory/inhibitory (E/I) balance have been hypothesized in idiopathic ASD and SSD [67, 69, 70]. Studies in LgDel/+ and Dgcr8+/− mouse models found dysregulated sodium potassium cotransporter protein expression (NKCC1 and KCC2) in hippocampal embryonic neuronal cultures, as well as disruptions in homeostatic synaptic plasticity that impacted the typical developmental E/I GABA polarity switch [71]. A recent study in postmortem tissue from SSD patients found regional shifts in the expression of cortical excitatory and inhibitory transcripts [72] and a previous study found alterations in regulators of NKCC1 and KCC2 [73], while animal models of other ASD-relevant gene mutations have also reported shifts in E/I balance [70]. The LgDel/+ model showed that gene dosage deficits affecting superficial layer 2/3 (but not layer 5/6) projection neurons resulted in cognitive impairment relative to wild-type mice [56]. Importantly, rescue of the neurobiological impact of this deletion early in development reversed the cellular, molecular, and behavioral deficits observed. Together, these findings suggest that discoveries in animal models of 22q11DS liability may index cellular and circuit mechanisms that could also underlie aspects of disease pathology in SSD and ASD.

**Fig. 2:**
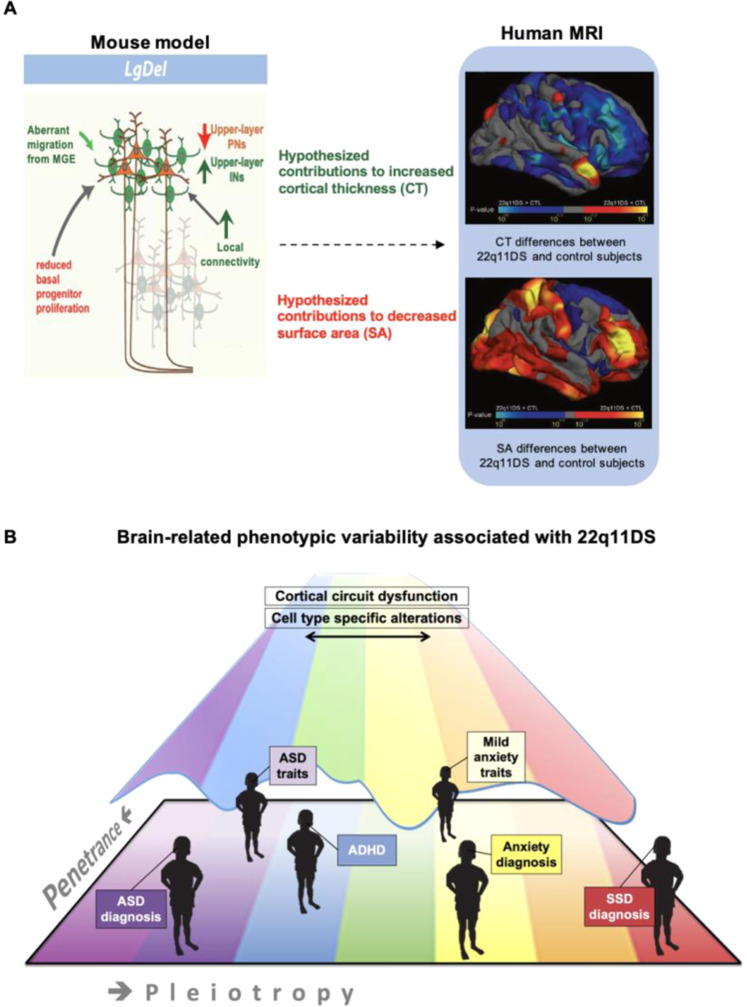
Select neurobiological alterations may underlie psychiatric heterogeneity in 22q11.2 deletion syndrome. To reduce complexity, one model animal system and imaging modality are shown to demonstrate neurobiological correlates of psychiatric phenotypes in 22q11.2 deletion syndrome. **A** Left panel shows a recent elegant molecular study in a 22q11.2 mouse model. Superficial (layer 2/3) pyramidal and GABA neurons are shown in focus in red-orange and green colors, respectively. These neurons had widespread alterations that were not seen in the deeper layer neurons (faded colors). Alterations in green may underlie, at least in part, cortical thickness increases in 22q11DS human subjects observed on structural MRI (right panel). Blue colors indicate greater cortical thickness (CT) in 22q11DS versus controls. Alterations in red may index, at least in part, surface area (SA) decreases in 22q11DS human subjects on structural MRI (right panel). Red colors indicate lower SA in 22q11DS versus controls. The dashed arrow indicates that these are hypothesized contributors across modalities and species, and that further studies are needed to elucidate many unanswered questions linking these associations mechanistically. **B** Distributed alterations in cortical structure and function in humans are hypothesized to contribute to a range of brain-related phenotypes and psychiatric disorders in 22q11DS, resulting in pleiotropy of diagnostic labels and intermediate traits, as well as a continuum of severity for each of these. ADHD attention-deficit/hyperactivity disorder, INs interneurons (GABA neurons), MGE medial ganglionic eminences, PNs projection (pyramidal) neurons, Adapted from [77, 156]).

The observed laminar, cellular, proliferation, and neuronal migration-related alterations in the LgDel/+ mouse model offer indirect but important neurobiological insights that can help understand neuroanatomic findings in patients with 22q11DS. For example, the radial unit hypothesis is an explanatory framework for cellular mechanisms underpinning changes in surface area and cortical thickness [74]. Evidence supporting this hypothesis shows that cortical surface area depends on the rate of neuronal stem cell proliferation and/or programmed cell death, while cortical thickness is governed by the neuron number per radial unit [75]. Findings in the LgDel/+ mouse model suggest that reduced basal progenitor proliferation and projection neurons, possibly due to early apoptosis, may be some cellular mechanisms underlying prominent cortical surface area *decreases* seen in structural MRI in people with 22q11DS (Fig. 2A). In contrast, aberrant migration of cortical GABA neurons from the medial ganglionic eminences, resulting in increases in superficial layer GABA neurons and increased local connectivity [76, 77], may contribute to *increased* cortical thickness in human 22q11DS (Fig. 2A).

#### In vitro models

In the past decade, human skin (fibroblast) and blood (monocyte) cells from patients with neurodevelopmental and psychiatric disorders [78, 79], including subjects with 22q11DS [52, 80] have been reprogrammed into *induced pluripotent stem cells* (iPSCs) that can be further differentiated into 2D brain cells or 3D organoids for in vitro study [81]. IPSCs provide unprecedented modeling potential since fibroblasts and monocytes can be easily harvested from subjects with 22q11DS and typically developing matched control subjects. Using iPSCs derived from eight patients with schizophrenia or schizoaffective disorder and 22q11.2 deletion and seven controls, one study showed differentially expressed genes particularly in MAPK signaling, cell cycle and apoptosis in the patient-derived iPSCs [80]. Using gene expression data from BrainSpan [82], the authors found that the differentially expressed genes in 22q11DS iPSCs converged on a CDC45-mediated cell cycle pathway involved in embryonic brain development and a PRODH-modulated network implicated in adolescent brain function [80]. The PRODH finding is intriguing in light of several studies showing that a subset of individuals with 22q11DS have increased levels of the principal substrate of PRODH, Proline [83], which was associated with brain function phenotypes in several studies [84–87]. A more recent, larger study using iPSCs to create 2D cell lines and 3D organoids from 15 subjects with 22q11DS and 15 matched controls found transcriptional changes in 22q11DS neurons enriched for genes that increase risk for ASD and SSD. Cellular phenotypes suggesting perturbed neuronal excitability at the transcriptional and electrophysiological levels—without gross deficits in corticogenesis—were observed, recapitulated by heterozygous *DGCR8* loss, and normalized by *DGCR8* rescue. Despite variable clinical phenotypes in the 22q11DS group, cellular phenotypes were robust and reproducible across subjects and cell lines, suggesting potential convergence at the level of molecular and cellular phenotypes [52].

While iPSC models are promising, there are important limitations [81]. For example, while 3D cultures, or organoids, are compelling models to study early stages of development, sample sizes remain small, raising concerns about statistical robustness and replicability. Technical considerations such as batch and cell-line variability have improved recently, but remain challenging confounds [88]. Another important consideration is identifying the most meaningful phenotypes and appropriate cell types to study in the context of a neuropsychiatric condition, given the lack of pathognomonic cell and molecular findings in psychiatric disorders. Identifying appropriate cell types remains challenging since iPSC models currently lack full neuronal and glial diversity [81]. This finding also raises the question of how well iPSC studies can model neural circuits, which is important given the relevance of neural circuit dysfunction in psychiatric disorders like SSD and ASD. Finally, while progress is being made in growth and maturation of iPSCs, they remain relatively immature [89].

In concert, the findings discussed throughout this section suggest that convergent molecular findings in 22q11 hemizygous deletion models may reveal genetic mechanisms of developmental cell-type and circuit-specific dysfunction underlying ASD or SSD symptoms. These model system studies also offer potential molecular explanations for structural MRI alterations observed in human 22q11DS. However, to date, studies of cellular models have not included sufficient numbers of subjects to convincingly demonstrate molecular causes of the observed phenotypic variability. This is an area of rapidly advancing technology; in future studies, models of 22q11.2 gene dosage alterations resulting in different neuropsychiatric phenotypes are likely to further our understanding of the biological subtypes of ASD or SSD, thereby providing valuable venues for the discovery of novel therapeutic interventions for these disorders.

## Understanding phenotypic variability

**Fig. 3:**
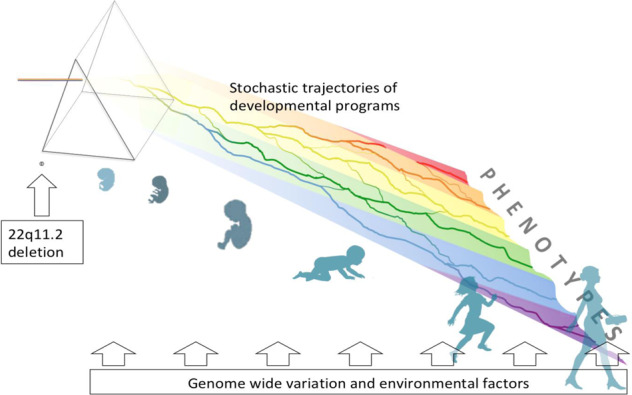
High-impact genetic variants such as the 22q11.2 deletion are thought to alter brain developmental programs. The resulting (range of) phenotypic outcomes in carriers of such variants are probabilistic due to the stochastic trajectories of developmental programs, analogous to the course of water flowing down a hilly landscape; trajectories of brain development are further influenced by additional genetic and environmental factors. The result of the interplay of these factors leads to variable phenotypic manifestations associated with the primary genetic variant, as represented by the color spectrum.

Even with a high-impact variant as a starting point, the picture is complicated by neuropsychiatric pleiotropy, as well as variable penetrance and expressivity (Table 1). These three phenomena, now considered common features of most pathogenic genetic variants associated with neurodevelopmental outcomes [26, 90], pose significant challenges to both research and clinical practice. A key question therefore is to understand the underlying mechanisms driving these phenomena. These likely include genetic variation in the rest of the genome, encompassing the modifying impact of additional rare variants [91] and the aggregate effects of common variants [92], environmental effects, and variation resulting from stochastic events during brain development, to whose influence individuals with high-impact variants may be particularly susceptible [19, 93]; Fig. 3). The combination of pleiotropy and variable penetrance means that some individuals with 22q11DS function in the average cognitive range, and without any psychiatric disorder, while others are severely affected by psychiatric multi-morbidity and/or cognitive impairment. A first step towards a better understanding of this phenotypic variability is to elucidate associations between the different phenotypic presentations.

### Pleiotropy

Pleiotropy, a phenomenon invariably observed in individuals with rare, pathogenic variants such as 22q11DS, often extends across multiple organ systems, but even when restricting to neuropsychiatric phenotypes, the range of pleiotropy is large. In 22q11DS, the neuropsychiatric phenotype alone includes varying levels of cognitive impairment and learning difficulties, language disorder, ASD, ADHD and anxiety disorders, schizophrenia and, later in life, Parkinson’s Disease [1, 3, 8].

Given the markedly increased rates of both ID and psychiatric disorders in individuals with 22q11DS, a salient question is to what extent these phenotypes are associated. In the general population, the overall rate of psychopathology is increased in youth with (idiopathic) intellectual impairment compared to youth with cognitive abilities in the average range [94]. Reported profiles of psychopathology in individuals with 22q11DS compared to IQ-matched individuals without 22q11DS suggest that the 22q11.2 deletion elevates the risk of some psychiatric disorders (e.g., SSD, ASD, ADHD, and anxiety disorders), but not others (e.g., disruptive and substance use disorders) [3, 8]. In addition, with the exception of SSD, studies have not found correlations between cognitive level and risk of psychiatric disorders in individuals with 22q11DS [95–98]. Collectively, this implies that the increased psychopathology risk in 22q11DS cannot be considered a non-specific consequence of ID, and that ID and psychiatric disorders may occur as—at least partly—distinct consequences of the 22q11.2 deletion [3].

For SSD, the picture is somewhat different: Both low (baseline) IQ and a decline in cognitive functioning have been associated with increased risk of SSD in individuals with 22q11DS, with the strongest effect size for a decline in Verbal IQ [3, 99, 100], largely converging with findings for idiopathic schizophrenia [101–103]. Thus, while cognitive decline and SSD were initially conceptualized as independent (pleiotropic) phenotypes in 22q11DS [11, 104], substantial evidence has accumulated that these phenotypes are at least partly associated and may represent different developmental stages of the same disease process [99, 100]. These findings in 22q11DS support the possibility of a similar association between early cognitive decline and schizophrenia proposed in the general population [105]. Further exemplifying how observations in 22q11DS can provide valuable insights for our understanding of disease mechanisms in idiopathic cases, an additional study reported a significant association between the polygenic score for schizophrenia and cognitive decline in 22q11DS, thus indicating that the modification of these phenotypes in 22q11DS occurs at least partly under the influence of the same common genetic risk variants [92].

**Fig. 4:**
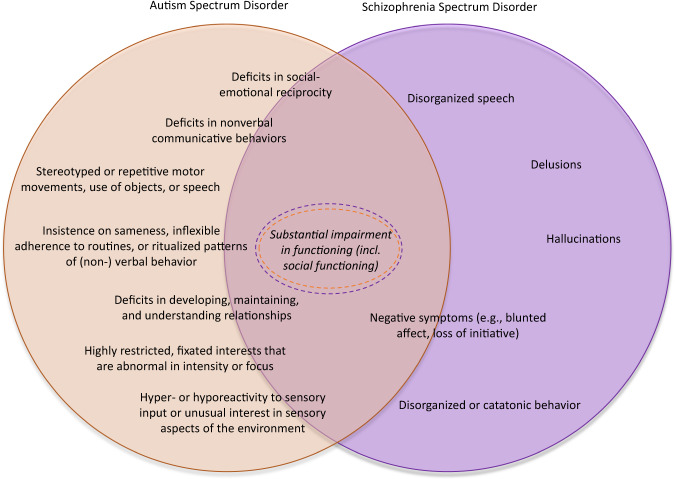
Symptom overlap between ASD and SSD. While the developmental timecourse of these diagnostic groups differs, specific symptoms and traits (indicated by the overlapping circles) are common to both. For some symptoms (e.g., blunted affect), it is difficult to determine whether they reflect a deficit in nonverbal communication vs. negative symptoms. Others (e.g., delusions) are unique to SSD vs. ASD.

The study of ASD and SSD in 22q11DS offers another window into neuropsychiatric pleiotropy and also highlights the importance of studying longitudinal trajectories of symptoms, as has been previously noted [106]. Rates of both ASD and SSD are elevated in 22q11DS [8, 107]. Similarly, in the general population, the association between ASD and SSD is complex [108], with substantial overlap between the two phenotypes [109] **(**Fig. 4**)**, increased rates of comorbidity among them, and accumulating evidence that individuals with ASD have an elevated risk for later SSD [110–112]. Indeed, both in the general population and in 22q11DS, the psychosis prodrome includes behaviors such as social withdrawal and increased communicative difficulties [113]; both of which are among the core symptoms of ASD. However, two independent studies have found no association between childhood autistic behaviors and subsequent psychosis risk in individuals with 22q11DS [114, 115].

These findings imply that in this genetic high-risk population, childhood autistic behaviors cannot be viewed as a clinical marker predictive of psychosis onset, and second, they indicate that ASD and SSD represent two distinct, largely independent phenotypes of a 22q11.2 deletion, with likely different underlying neurobiological pathways, indicative of neuropsychiatric pleiotropy.

The association between ASD, an early-onset phenotype, and schizophrenia, which typically has overt symptom onset in early adulthood, is challenging to study in the general population. Findings in 22q11DS [114, 115] suggest that the increased comorbidity between these diagnoses in the general population [116] may be partly explained by high-impact pathogenic variants, which increase risk for both conditions. Taken together, such findings may prompt further investigations both between and within groups of individuals with such genetic variants, to elucidate the potential specificity of genetically determined “subtypes” of ASD, and how these may relate to psychosis risk.

In addition, the phenotypic manifestations of schizophrenia in the context of a 22q11.2 deletion, including subthreshold [117] and overt clinical symptoms, illness trajectory, and response to treatment, appear comparable to those of idiopathic schizophrenia [118]. However, it is plausible that—similar to the observations for ASD—some characteristics of SSD may be specific to the underlying genetic contribution. For example, some studies suggest there may be an earlier age at onset of schizophrenia in 22q11DS compared to idiopathic SSD [100]. Indeed, the rate of 22q11DS in childhood-onset schizophrenia was found to be significantly higher than that in adult onset illness [119]. At present, more research is warranted to determine whether age of psychosis onset is truly earlier in 22q11DS, due to higher genetic burden [120] and biological vulnerability [121, 122], or a function of earlier detection due to ascertainment bias.

### Variable penetrance and expressivity

Even though neuropsychiatric disease risk is greatly increased in 22q11DS compared to the general population, the majority of individuals with 22q11DS (~75%) do not develop SSD, and 60-90% do not meet diagnostic criteria for ASD [8]. Such incomplete penetrance is not only the rule for all 22q11DS-associated phenotypes (e.g., ~25% of individuals with 22q11DS do not have a congenital cardiac anomaly [1]), it is also the prevailing phenotypic pattern observed for virtually all other rare, high-impact variants [123]. From a research perspective, it begs the question what additional factors modulate the phenotypic outcome in any given individual. Clinically, variable penetrance hampers our ability to provide robust clinical predictions for individual patients. However, in addition to its traditional categorical conceptualization, penetrance can also be conceptualized dimensionally. Cognition as a phenotype can illustrate both approaches in 22q11DS: the penetrance of ID—defined categorically as IQ below 70—is estimated to be around 45–50% [124]. From a dimensional perspective, the distribution of IQ in 22q11DS, with an average of 70, is shifted approximately 30 points (or 2 standard deviations) to the left compared to the general population [99]. Similarly, while the penetrance of SSD and ASD diagnoses in 22q11DS is 25% and 10–40%, respectively, various studies have observed that the proportions of individuals with subclinical manifestations of both conditions are substantially higher [107, 125]. In addition, ascertainment strategies may bias recruitment, potentially towards the severe end of the phenotypic spectrum, in clinical studies.

These examples illustrate how the concept of ‘penetrance’ of a trait (as defined by dichotomous phenotypes) may hinder more complete understanding of the full breadth of phenotypic consequences related to a genetic risk variant. For example, population-based and family studies support a dimensional view of autistic-like traits, suggesting that a categorical ASD diagnosis represents the extreme tail end of a continuous distribution [126]. This is consistent with the Research Domain Criteria (RDoC) approach advanced by the National Institute of Mental Health (Table 1; [127]. Investigating this question in the context of autism-related behavior in 22q11DS, we previously found that the best-fitting model for most neurocognitive measures was a dimensional one; in contrast, for a neuroimaging trait (bilateral parahippocampal thickness), the best-fitting model emerged when categorical diagnosis of ASD was used as a predictor [128]. These results highlight the complexity of the problem, and suggest that a combination of dimensional and categorical variables may offer the most comprehensive understanding of ASD symptomatology in patients with 22q11DS.

### The modifying role of additional genetic variation

Recently, findings are emerging that shed light on the role of common genetic variation in the context of a high-impact genetic variant such as the 22q11.2 deletion. Specifically, studies from the 22q11DS international Brain and Behavior Consortium (iBBC) [129] showed significant associations of the cumulative impact of common variation associated with schizophrenia and IQ, summarized as polygenic scores, with these respective phenotypes in 22q11DS [92, 130]. These observations confirm that additional genetic variation modifies the nature and severity of neurodevelopmental phenotypic expression, i.e., pleiotropy and penetrance [131, 132]. A potential clinical application of these observations is that polygenic risk score findings may become useful for outcome prediction in patients with rare high-impact pathogenic variants. In such individuals, the same genetic background factors influence risk for neurodevelopmental outcomes as in the general population. However, due to the elevated a priori risk conferred by the primary genetic variant, a much larger proportion will develop the phenotype [18]. Current empirical evidence supports this mechanism for the phenotypes of IQ and schizophrenia, but it is not yet known if this pattern generalizes to other brain-related phenotypes. A recent study of cognitive, social, and motor phenotypes in individuals with a *de novo* 22q11.2 deletion and their unaffected parents found a significant association between parental and offspring cognitive functioning, consistent with the polygenic score findings [133].

### Influences of environment and intrinsic developmental variation

In addition to the emerging importance of genomic context in which pathogenic variants exist, initial evidence suggests that environmental and stochastic developmental factors also shape phenotypic outcomes in individuals with a high-impact genetic variant such as 22q11DS [17, 18], analogous to how they impact outcomes in the general population. Findings on environmental influences in the context of high-impact variants are currently scarce, but they provide direction for future investigations.

Both macro- and micro-level environmental factors (low parental socio-economic status and intrusive parenting style, respectively) have been associated with worse social functioning and other clinically significant problems in children with 22q11DS [134, 135]. In addition, there is some evidence that stressful life events may modulate the risk for psychotic symptoms in adolescents with 22q11DS, and that individuals with 22q11DS may have a differential stress response [136, 137]. A recent study reported an association between parental anxiety and depression with offspring’s psychopathology, which was, notably, stronger in the 22q11DS group compared with a typically developing control group [138]. Here, again, the high a priori risk in 22q11DS may act as a magnifying lens with regard to such mechanisms. While not directly tested in the study, the authors note that these findings likely reflect both gene*environment (G*E) effects, as well as gene*gene (G*G) effects. This notion may be generalizable to the effects of *any* environmental factor, and is congruent with the proposition of genetic nurture, reflecting that parental genetic background, even if not transmitted to offspring, can impact offspring phenotypic expression by, for example, shaping parenting style [139]. This *nature of nurture* effect, reflecting that our genetic background shapes our environments, poses challenges for identification of environmental markers associated with phenotypic outcomes, even in the context of high-impact genetic variants.

Further, stochastic influences introduce liability for one or several neurodevelopmental phenotypic outcomes [93] (Table 1 and Fig. 3). In addition, the degree of impact of any stochastic event may be variable, and a consequence of developmental robustness, potentially a (genetic) trait in and by itself [140]. It is likely that the presence of a high-impact variant such as the 22q11.2 deletion lowers this developmental robustness, leaving the developmental trajectories of individuals with 22q11DS more susceptible to the impact of stochastic events.

### Animal models: genetic background, pleiotropy, and phenotypic variation

Mouse models of 22q11.2 haploinsufficiency with controlled genetic backgrounds may help disentangle the role of genetic variation in the rest of the genome versus phenotypic variability introduced by the deletion itself [38, 59]. For example, the modifying effect of genetic background was reflected via social interaction phenotypes in SEPT5 knockout mice, which were only apparent in congenic mice [141, 142]. The loss of phenotypic expression might be due to a modifying impact of the congenic mice alleles on the SEPT5 mutation [59]. Numerous other congenic or coisogenic mouse models (Table 1) have been developed to examine the impact of deletions of individual 22q11.2 genes on various behavioral constructs relevant to psychiatric disorders [55, 143–149].

Even when genetic background is controlled, mice with deletion of murine homologs of the human 22q11.2 genes have shown different phenotypes. For example, LgDel/+ mice had impaired reversal learning while Df(h22q11)/+ mice had enhanced reversal learning, even though their mutations are fairly similar, with LgDel/+ mice having more protein-coding genes deleted and numerous backcrosses during breeding [150, 151]. Mouse studies manipulating expression of individual 22q11.2 genes suggest that antagonistic effects of genes within the 22q11.2 deletion may contribute to variable phenotypic effects of the deletion itself. For example, *Tbx1* mutant mice have impairments in working memory, social behaviors, and anxiety-like behaviors that are consistent with human 22q11DS [152, 153], whereas *Comt* mutant mice showed either no effect on prepulse inhibition (PPI), social behaviors, and anxiety-like behaviors, or effects on working memory inconsistent with human 22q11DS [143, 146–149]. As reviewed in Hiroi [38], mouse models for other individual 22q11.2 genes have been tested in various behavioral tasks, but no differences in phenotypes tested were found relative to wild-type littermates after controlling for genetic background [38]. Together, these findings raise the possibility that the collective action of multiple 22q11.2 genes determines the behavioral phenotype, consistent with the concept of a contiguous deletion syndrome, and that some genes deleted in 22q11DS may have antagonistic effects, all of which likely influence phenotypic expression.

Animal models of 22q11DS liability also support the concepts of pleiotropy and phenotypic variation observed in human subjects with 22q11DS. For example, Df1/+ mice have deficits in sensorimotor gating and psychostimulant induced hyper-locomotion, as well as motor coordination deficits, elevated alpha synuclein and p62 proteins in multiple brain regions, showing pleiotropy for SSD and Parkinson’s disease-relevant phenotypes (Table 1; [154]. Heterozygous knockout of individual genes within the locus also results in some overlapping, pleiotropic phenotypes: for example, *Tbx1* hemizygosity causes deficits in working memory, social interaction and communication, as well as repetitive behavior tendencies, phenotypic traits commonly observed in human ASD [153]. *Zdhhc8* deletion affects both prepulse inhibition and anxiety-related behavior [155], while *Dgcr8* deletion lowers scores for both prepulse inhibition and working memory [43].

Additionally, variable cortical alterations may be related to particular outcomes in individuals with 22q11DS (Fig. 2). For example, Sun et al. [156] found that 22q11DS subjects with psychosis showed significantly thinner cortex in predominantly frontal and temporal brain regions compared to those without psychosis. Another study, comparing the brains of youth with 22q11DS with and without ASD to idiopathic ASD and typically developing controls, found that the main effect of 22q11DS was distinct from the neuroanatomical underpinnings of the main effect of ASD [157]. These two examples show unique neurobiological signatures in 22q11DS with SSD or ASD [128]. An important caveat regarding human brain-behavior association studies presently is that large sample sizes are required to generate reproducible results due to sampling variability, effects of image acquisition on different scanners and data processing variability [158]. Therefore, interpretation of neuroimaging studies to date should consider these limitations. Nevertheless, in this context it is also important to note that highly penetrant CNVs like the 22q11.2 deletion have much more robust and consistent effects on brain phenotypes than idiopathic psychiatric disorders and those observed in typical development [156, 159, 160].

These findings suggest that haploinsufficiency for one or more genes in the 22q11.2 locus may result in multiple heterogeneous phenotypes, depending on the genetic background and environmental factors during development. Furthermore, recent molecular studies support the hypothesis that there may be shared final common neural circuit pathways where polygenic and poly-environmental factors converge before emergence of multiple, distinct phenotypes [76, 154, 161]. Understanding where and how convergence arises between genetic variation and neurodevelopmental processes, and how this relates to the distinct psychiatric manifestations associated with certain pathogenic risk variants, remains a challenge for the field.

## Conclusion

This review focuses on findings from studies of the 22q11.2 deletion, a particularly compelling and well-characterized example of a highly penetrant genetic variant. In particular:

(1) We advocate for a conceptual shift, moving from an exclusively descriptive basis of psychiatric classification to an integrative approach that makes explicit the connection between the psychiatric and genetic diagnosis (e.g., “schizophrenia, related to 22q11.2 deletion”). While we focus on 22q11DS specifically, the findings are relevant to the many other rare pathogenic variants associated with substantial risk for neurodevelopmental and psychiatric outcomes. This is increasingly important given that the number of such variants is steadily rising, imposing a growing impact on clinical practice [17]. From this perspective, the proposed integrative diagnostic approach is not limited to 22q11DS, but is also relevant to other genetic conditions (e.g. “ADHD, related to 7q11.23 duplication” [33]). As our insights into the association between genetic etiologies and neuropsychiatric phenotypes continue to evolve, studies should continue to investigate how to further implement a neuropsychiatric classification, which integrates the latest genetic knowledge wherever possible.

(2) Studies of 22q11DS highlighted here exemplify research approaches that capitalize on the premise of increased homogeneity when stratifying subjects with neuropsychiatric phenotypes such as ASD or SSD by shared genetic basis [59]; further investigations into potential “genetic subtypes” of ASD and SSD are warranted. Clinical ramifications include etiologically specific neurodevelopmental trajectories with opportunities for targeted early intervention and, potentially, the development of genetically guided pharmacological interventions [29, 162]. For example, Angelman syndrome is a rare neurodevelopmental disorder involving severe developmental delay, intellectual disability and seizures, resulting from loss of function of the maternally inherited *UBE3A* gene on chromosome 15q11–13 [163]. Emerging small molecule drug and gene therapies involve restoration of *UBE3A* function [164]. While multi-gene CNVs like 22q11.2 obviously present greater challenges for such treatments than do single-gene disorders, there is nevertheless exciting potential for gene-editing therapies, e.g., that could up-regulate the intact allele [165].

(3) Variable penetrance and pleiotropy of neuropsychiatric phenotypes are not unique to 22q11DS, but are clearly the rule rather than the exception for the majority of rare pathogenic variants. Here, again, findings in 22q11DS may provide helpful guidance for our understanding of the mechanisms underpinning these same phenomena in the context of other genetic variants. As discussed here, these likely include genetic variation in the rest of the genome, encompassing the modifying impact of both rare and common variants [98, 99], environmental effects, and variation resulting from stochastic events during brain development. Animal models of 22q11DS have indeed shown that genetic background, as well as environmental and stochastic developmental effects modulate phenotypic expression and have pleiotropic effects [40, 109, 166–168]. Recent studies in individuals with 22q11DS corroborate these findings for the impact of genetic background, providing new insights into the genetic architecture underlying enhanced disease risk in the context of a ‘neuropsychiatric’ CNV [91, 92]. Future studies designed to understand how these complex factors modulate phenotypic expression and pleiotropy via neural circuit and cell-type specific mechanisms, at specific developmental timepoints, are needed. Such work is imperative to develop more individualized risk prediction and treatment planning in the clinic for 22q11DS and beyond.
